# The 2024 Public Health Emergency of International Concern: A Global Failure to Control Mpox

**DOI:** 10.4269/ajtmh.24-0606

**Published:** 2024-10-15

**Authors:** Alimuddin Zumla, Philip J. Rosenthal, Nada A. Sam-Agudu, Dimie Ogoina, Placide Mbala-Kingebeni, Francine Ntoumi, Emmanuel Nakouné, Richard Njouom, Nicaise Ndembi, Edward J. Mills, Jean-Jacques Muyembe-Tamfum, Jean B. Nachega

**Affiliations:** ^1^ Division of Infection and Immunity, Centre for Clinical Microbiology, University College London, United Kingdom;; ^2^ National Institute for Health and Care Research (NIHR) Biomedical Research Centre, University College London Hospitals NHS Foundation Trust, London, United Kingdom;; ^3^ Department of Medicine, University of California San Francisco, California;; ^4^ International Research Center of Excellence, Institute of Human Virology Nigeria, Abuja, Nigeria;; ^5^ Department of Paediatrics and Child Health, University of Cape Coast School of Medical Sciences, Cape Coast, Ghana;; ^6^ Global Pediatrics Program and Division of Infectious Diseases, Department of Pediatrics, University of Minnesota Medical School, Minnesota;; ^7^ Department of Internal Medicine, Infectious Diseases Unit, Niger Delta University and Niger Delta University Teaching Hospital, Bayelsa, Nigeria;; ^8^ Institut National de la Recherche Biomédicale (INRB), Kinshasa, Democratic Republic of the Congo;; ^9^ Fondation Congolaise de la Recherche Medicale (FRCM), Brazzaville, Republic of Congo;; ^10^ Institute of Tropical Medicine, University of Tübingen, Tübingen, Germany;; ^11^ Centre Pasteur du Cameroun, Unité de Virologie, Yaoundé, Cameroun;; ^12^ Institut Pasteur de Bangui, Bangui, Central African Republic;; ^13^ Africa Centres for Disease Control and Prevention (Africa-CDC), Addis-Ababa, Ethiopia;; ^14^ Department of Health Research Methods, Evidence and Impact, Faculty of Health Sciences, McMaster University, Hamilton, Ontario, Canada;; ^15^ Department of Medicine, Division of Infectious Diseases, Stellenbosch University Faculty of Medicine and Health Sciences, Cape Town, South Africa;; ^16^ Departments of Epidemiology and International Health, Johns Hopkins Bloomberg School of Public Health, Baltimore, Maryland;; ^17^ Departments of Epidemiology, Infectious Diseases, and Microbiology, University of Pittsburgh School of Public Health, Pittsburgh, Pennsylvania

## Abstract

On August 14, 2024, following a regional declaration by the Africa Centres for Disease Control and Prevention, the World Health Organization declared mpox a Public Health Emergency of International Concern, marking the second such declaration in two years. A series of outbreaks involving the more virulent clade I virus (compared to clade II, which caused a global outbreak in 2022), has now spread in 13 African countries, exposing the inadequacies of the public health infrastructure in these settings. There was significant investment during the 2022 global outbreak, but these efforts failed to address vaccine access and treatment in the Global South. Regulatory delays, unequal access to vaccines, and a lack of compassionate use treatments for severe cases have resulted in preventable cases and deaths, especially among vulnerable populations such as pregnant women, children, and the immunocompromised. The current outbreak also underscores critical knowledge gaps in our understanding of mpox, including its transmission, pathogenesis, and viral evolution. We join intensified calls for global solidarity and action to control mpox, emphasizing immediate containment measures and long-term local and international investment in African public health systems, to prevent future epidemics.

On August 14, 2024, the World Health Organization (WHO) declared the current mpox outbreak a global Public Health Emergency of International Concern (PHEIC).^1^ This marks the second mpox PHEIC declared by the WHO in the past two years, which followed the regional emergency declaration by the Africa Centres for Disease Control and Prevention (Africa CDC) on August 13, 2024.^2^ These declarations expose major gaps in mpox vaccine and drug access, as well as in understanding the natural history of the disease, transmission dynamics, pathogenesis, viral evolution, immune responses, and underlying risk factors that fuel outbreaks and sustain transmission.

Despite the WHO’s initial PHEIC declaration on July 23, 2022, which was prompted by mpox spreading outside of Africa, the continent continues to be disproportionately affected. The first mpox PHEIC officially ended on May 11, 2023, yet the challenges in addressing mpox in Africa persist. Prior to the global 2022 outbreak, the majority of mpox cases were concentrated in the Democratic Republic of Congo (DRC). However, limited access to diagnostics, therapeutics, and vaccines has left African nations vulnerable to successive outbreaks. There are two major genetic clades of monkeypox virus (MPXV): clade I (formerly known as Central African or Congo basin clade) and clade II (formerly known as West African clade). Clade I (case fatality ratio (CFR) up to 10%) is categorized into Ia and Ib, and clade II (CFR <3%) into IIa and IIb, with subgroup clusters called lineages. Most of the cases seen in the global 2022 outbreak were clade IIb infections, primarily reported among men who have sex with men, with a relatively low CFR (<0.2%).^3^

Recent reports have raised concerns about an outbreak of clade I MPXV in the DRC; in 2023 alone, more than 12,000 cases and 600 deaths were reported to the WHO.^2^^–^^5^ From January 1 to September 23, 2024, 13 African countries have reported 35,341 cases of mpox (3,331 confirmed; 32,010 suspected) and 840 deaths.^5^ The Africa CDC intelligence service weekly report of September 23, 2024, documented mpox clade I from the DRC (5,599), Burundi (564), Central Africa Republic (52), and Uganda (22). The Mpox Research Consortium (MpoxReC)^6^ has identified Kamituga, a gold-mining town in Eastern DRC, as a hotspot, with 241 cases reported between September 2023 and January 2024. The median age for cases in Kamituga was 22 years, with 52% of cases in women and 29% among sex workers, suggesting sexual transmission. Genomic analysis identified Clade Ib6 with APOBEC3-type mutations, indicating sustained human transmission.^7^ The spread of Clade Ib infections beyond the DRC to neighboring countries, and beyond, coupled with re-emergence of Clade IIb outbreaks in Nigeria and South Africa, prompted the WHO 2024 PHEIC declaration. Children and adolescents are the age group most affected by mpox. Nearly 70% of the 7,851 PCR-confirmed mpox cases reported to the WHO by the DRC Ministry of Health from January to May 2024 occurred in patients <15 years old, including 321 deaths (83% of total fatalities).^6^ Alarmingly, the highest mortality rate was observed among infants <1 year old (CFR 8.7%), with an odds ratio of death 3.8 times higher than that in those ≥15 years old (CFR 2.4%).^6^ This difference highlights the severe impact of mpox on young children.^4^^,^^5^

The prior global clade IIb outbreak stimulated substantial, rapid investment in diagnostics, vaccines, and therapeutics in the Global North.^8^^,^^9^ Also, the declarations of Ebola and COVID-19 as PHEICs led to investments to establish the Africa CDC, the development of African public health surveillance and laboratory infrastructure, and the convening of multidisciplinary, international networks focused on epidemic preparedness in Africa.^10^ However, the recent mpox outbreak is an indication that these efforts were insufficient to prevent repeated mpox outbreaks across the continent. Furthermore, the effectiveness of public health interventions alone (such as contact tracing, isolation, and quarantine campaigns) without targeted medical countermeasures in controlling clade I outbreaks is not well documented, particularly in resource-limited settings where most clade I cases occur.

Following prior international outcries after outbreaks of Ebola, COVID-19, and mpox, expectations of a major paradigm shift in global vaccine equity and health systems strengthening have not yet been realized in Africa.^11^ It was anticipated that significant investment would be made to build robust infrastructure to prepare for, and respond to, future pandemics, including rapid vaccine production.^12^ West/Central African governments and the Africa CDC proactively joined together to improve health systems for the mpox response; however, the need for another recent emergency declaration highlights persistent inadequacies. The new WHO PHEIC declaration presents an opportunity to focus global dialogue on key issues concerning not only mpox, but epidemics in general. African countries continue to face shortages of diagnostics, vaccines, and therapeutics for several infectious agents. For example, current pledges and donations for mpox vaccines fall significantly short of the estimated 20 million doses needed to control the spread of mpox in endemic African countries. This shortfall exemplifies the global failure to deliver on promises made during the COVID-19 pandemic to support Africa’s immediate and long-term vaccine needs and to develop local vaccine and therapeutics manufacturing capabilities.^13^^–^^15^

Due to the broad cross-immunity observed within the *Orthopoxvirus* family, the WHO Strategic Advisory Group of Experts on Immunization recommends two smallpox vaccines for mpox prevention: the Modified Vaccinia Ankara vaccine (MVA-BN) from Bavarian Nordic and the LC16m8 vaccine from KM Biologics.^16^ The MVA-BN vaccine, containing a live, non-replicating virus, is marketed as JYNNEOS in the Unites States. It is approved for individuals aged 18 years and older, including pregnant and breastfeeding women.^17^ The LC16m8 vaccine, approved in Japan and the DRC for both adults and children, is contraindicated for immunocompromised patients and during pregnancy because it contains a live, replicating attenuated virus.^18^ However, as efficacies of both vaccines were inferred from neutralizing antibody levels for smallpox, clinical trials are needed to confirm their effectiveness in preventing clade I and clade II MPXV infections among participants who do not have contraindications.^19^

As highlighted recently, bureaucratic and regulatory delays at the WHO have severely hindered distribution of the MVA-BN and LC16m8 vaccines.^20^ The DRC received its first vaccine delivery in early September 2024. This milestone was achieved through a partnership between Africa CDC, the European Union Health Emergency Preparedness and Response Authority, and Bavarian Nordic. However, this delivery totalled only about 200,000 of the 10 million doses estimated by Africa CDC for the control the DRC mpox epidemic.

With respect to therapeutics, there has been a failure to provide the antiviral drug tecovirimat for compassionate use in severely ill persons through the WHO Monitored Emergency Use of Unregistered and Investigational Interventions program.^21^ Notably, preliminary results from the U.S. National Institutes of Health co-sponsored PALM007 trial, evaluating tecovirimat efficacy against clade I MPXV, posted on August 15, 2024, indicated that the study did not meet its primary endpoint of statistically significant improvement in time-to-lesion resolution. However, sub-group analyses of this study are ongoing.^22^ There are other ongoing phase 3 trials evaluating tecovirimat for treatment of mpox, including the STOMP trial in the United States (NCT05534984); the PLATINUM trial in Canada and the United Kingdom (NCT05534165); the UNITY trial in Switzerland and Brazil (NCT05597735); and the TECOPOX trial in Japan (jRCTs031220169). Until more data are available, tecovirimat remains recommended for compassionate use in severe mpox cases, but there is an urgent need to explore alternative treatments.

The declaration of the second mpox PHEIC by the WHO is intended to rapidly mobilize global resources and focus international attention to combat this outbreak. Since PHEICs are temporary and require review every three months, African countries must act swiftly to capitalize on this declaration to facilitate in-country regulatory processes to access medical countermeasures (diagnostics, therapeutics, and vaccines) and secure adequate resources to control the outbreak and build sustainable infrastructure. Given the historically poor showing of commitment and action by the global community in previous emergencies, African countries should not wait for external assistance. African governments, institutions, and leaders should lead in funding and implementing coordinated mpox responses to prevent further spread and deaths across the continent. Recent laudable partnerships between philanthropic foundations, such as the Bill and Melinda Gates Foundation and the King Salman Humanitarian and Relief Foundation, which have supported polio vaccination programs, should now be extended to support efforts to control mpox and other vaccine-preventable diseases with epidemic potential, particularly in low- and middle-income countries.^23^^,^^24^ Jynneos vaccine availability to the region should also expand through partnerships with the Africa CDC, WHO, European Centre for Disease Prevention and Control, and the U.S. CDC.^25^^,^^26^

Between 2005 and 2023, the WHO declared eight PHEICs, namely H1N1 influenza (2009–2010); wild-type polio (2014–ongoing); Ebola (2013–2016 and 2018–2020); Zika neurologic syndromes (2015–2016); COVID-19 (2020–2023); and mpox (2022–2023 and 2024). Encouragingly, in contrast to the arguably delayed declaration of the COVID-19 PHEIC, the 2024 mpox declaration^1^ was comparatively quick and decisive, as was the Global Health EDCTP3 call for proposals, releasing emergency funding for mpox outbreak research.^27^ These were encouraging developments and may reflect a major step forward towards rapid, rational decision-making. Now, a paradigm shift from the global community needs to occur in response to PHEICs declared for pathogens that predominantly affect Africa. A prompt and decisive response to the new mpox outbreak by African nations and global partners will mark a welcome change in our approach to global outbreaks, epidemics, and pandemics.
